# Human Endometrial Stromal Cells Are Highly Permissive To Productive Infection by Zika Virus

**DOI:** 10.1038/srep44286

**Published:** 2017-03-10

**Authors:** Isabel Pagani, Silvia Ghezzi, Adele Ulisse, Alicia Rubio, Filippo Turrini, Elisabetta Garavaglia, Massimo Candiani, Concetta Castilletti, Giuseppe Ippolito, Guido Poli, Vania Broccoli, Paola Panina-Bordignon, Elisa Vicenzi

**Affiliations:** 1 Viral Pathogens and Biosafety Unit, Division of Immunology, Transplantation and Infectious Diseases, San Raffaele Scientific Institute, Milan, Italy; 2 Reproductive Sciences Laboratory, Division of Genetics and Cell Biology, San Raffaele Scientific Institute, Milan, Italy; 3 Division of Neuroscience, San Raffaele Scientific Institute, Milan, Italy; 4 Obstetrics and Gynecology Unit, San Raffaele Scientific Institute, Milan, Italy; 5 Vita-Salute San Raffaele University School of Medicine, Milan, Italy; 6 National Institute for Infectious Diseases “Lazzaro Spallanzani”, Rome, Italy; 7 AIDS Immunopathogenesis Unit, Division of Immunology, Transplantation and Infectious Diseases, San Raffaele Scientific Institute, Milan, Italy; 8 National Research Council (CNR), Institute of Neuroscience, Milan, Italy

## Abstract

Zika virus (ZIKV) is a recently re-emerged flavivirus transmitted to humans by mosquito bites but also from mother to fetus and by sexual intercourse. We here show that primary human endometrial stromal cells (HESC) are highly permissive to ZIKV infection and support its *in vitro* replication. ZIKV envelope expression was detected in the endoplasmic reticulum whereas double-stranded viral RNA colocalized with vimentin filaments to the perinuclear region. ZIKV productive infection also occurred in the human T-HESC cell line together with the induction of interferon-β (IFN-β) and of IFN-stimulated genes. Notably, *in vitro* decidualization of T-HESC with cyclic AMP and progesterone upregulated the cell surface expression of the ZIKV entry co-receptor AXL and boosted ZIKV replication by *ca.* 100-fold. Thus, endometrial stromal cells, particularly if decidualized, likely represent a crucial cell target of ZIKV reaching them, either via the uterine vasculature in the viremic phase of the infection or by sexual viral transmission, and a potential source of virus spreading to placental trophoblasts during pregnancy.

Zika Virus (ZIKV) is a member of the *Flaviviridae* family transmitted to humans by bites of the *Aedes* mosquito species^1^. The virus has been first isolated from the blood of a febrile monkey in 1947 in the Zika forest of Uganda^2^; however, its potential as a human pathogen was underestimated for almost 60 years until 2013 when an unsual outbreak of ZIKV-related Guillain-Barré syndrome emerged in French Polynesia^3^. A global health emergency was triggered at the end of 2015 by the observation of an increased incidence of microcephaly that was associated with the temporal and geographic distribution of ZIKV infection in the North East Brazil^4^. Increasing evidence now clearly supports a cause-effect relationship between congenital ZIKV transmission and increased frequency of mild to severe neuropathologies including microcephaly^5^,^6^. ZIKV was detected in the amniotic fluid of pregnant women^7^ suggesting that the placenta might be permissive to virus passage. This condition likely occurs during the first trimester of pregnancy^8^, although placental cells appear to be protected against ZIKV infection by a constitutive interferon (IFN)-λ1 response^9^. Indeed, three recent studies showed that human primary placental macrophages, trophoblasts and fibroblasts of the maternal *decidua basalis* were permissive to ZIKV productive infection *in vitro*^10^,^11^,^12^. Additional studies demonstrated that ZIKV can cross the placental barrier either via uterine vessels or by an ascending route as recently demonstrated in a mouse model of vaginal ZIKV infection^13^.

Vector-independent transmission of ZIKV among humans can indeed occur through the sexual route^14^. Male-to-female transmission has been reported in approximately 15 cases thus far^15^. ZIKV sexual transmission is also supported by both the detection of higher viral load in semen *vs.* blood and by the persistence of ZIKV in semen for several months after waning of symptoms^16^,^17^,^18^,^19^. More recently, female-to-male sexual transmission of ZIKV infection was also documented^20^. These observations imply a potentially prominent role of the female reproductive tract (FRT) as a site of virus infection and propagation either from and to the male partner during sexual intercourse or to the fetus during pregnancy. All compartments of the FRT, including the endometrium, might contribute to establishing and spreading the initial infection, during the initial viremic phase that is particularly prolonged during pregnancy^21^. In addition, it should be taken into consideration the fact that the human endometrium is a highly dynamic tissue undergoing major histological changes during the menstrual cycle under the coordinated action of sexual hormones. Estrogen dominates the proliferative phase of the menstrual cycle, while the post-ovulatory rise of ovarian progesterone drives the differentiation of human endometrial stromal cells (HESC) adjacent to spiral arteries^22^. This process, known as pre-decidualization, is critical for fetal trophoblast invasion and placenta formation and occurs independently of an implanting blastocyst^23^. Thus, decidualized perivascular stromal cells could be a potential target of circulating ZIKV. Furthermore, it is well documented that the progesterone-dependent secretory phase of the menstrual cycle represents a “window of opportunities” for several viral pathogens, such as HIV^24^ and herpes viruses^25^.

Therefore, we investigated whether primary HESC or immortalized (T-HESC) cells were permissive to *in vitro* ZIKV infection and replication. Indeed, ZIKV productively infected both HESC and T-HESC, whereas *in vitro* decidualization of the cell line (dT-HESC) increased both the expression of putative ZIKV entry co-receptor AXL and the levels of productive infection *vs.* unstimulated cells. Thus, our results suggest a relevant role of the endometrium in spreading ZIKV infection.

## Results

### ZIKV infection of primary HESC

**Figure 1:**
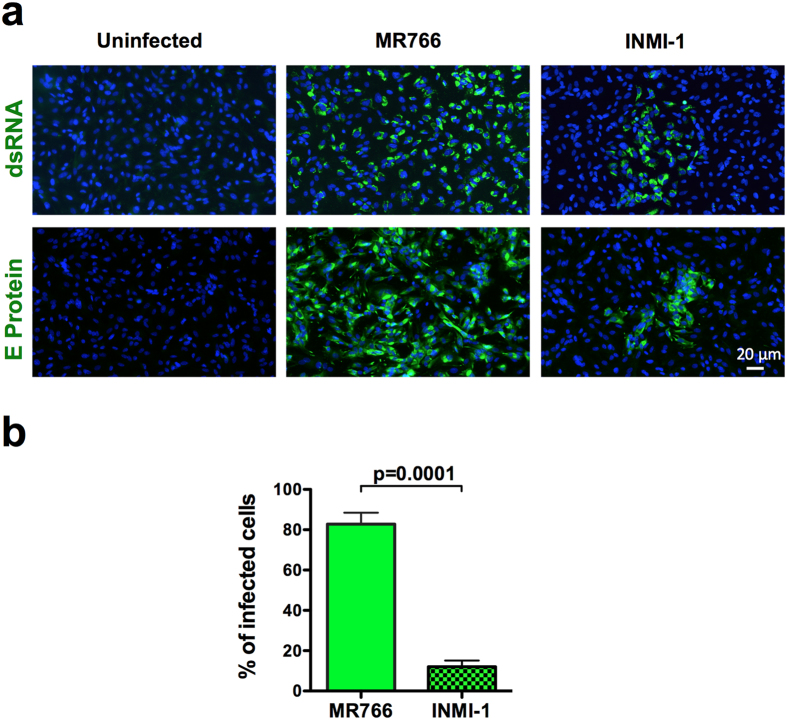
Primary HESC are permissive to ZIKV productive infection. (**a**) Immunostaining for ZIKV dsRNA and E protein of primary HESC infected with MR766 and INMI-1 ZIKV strains 72 h post-infection; Hoechst was used to stain nuclei. (**b**) Bar graph represents the mean ± SEM values determined from 10 fields of view for each donor (n = 2). P value was calculated by a paired Student’s t-test using the arcsine tranformation of the data.

Primary HESC were isolated from endometrial biopsies and incubated with either the reference African MR766 or contemporary INMI-1 strains at the multiplicity of infection (MOI) of 10 after reaching cell confluency (days 3–4). Viral growth was firstly analyzed by an indirect immunofluorescence assay (IFA) using either anti-ZIKV dsRNA or anti-ZIKV envelope (E) protein monoclonal antibodies (mAb). Subcellular distributions of both viral RNA and E protein were observed 72 h after infection of HESC (Fig. 1a). The percentage of cells infected with the MR766 strain, as measured by both anti-dsRNA and anti-E protein mAb staining, was *ca.* 80% whereas the proportion of cells infected with the INMI-1 strain was significantly lower (*ca.* 7%) (Fig. 1b). Although infection of primary HESC was established from 8 independent donors, as tissue availability was limited, only cells from three donors could be infected side-by-side with both viral strains. Nevertheless, as shown in Supplementary Fig. S1, all viral titers, but one, of MR766 infection were *ca.* one log_10_ higher than those obtained with the INMI-1 strain. Productive infection by two additional ZIKV strains, Puerto Rico 2015 (PRVABC59) and Thailand 2013^26^, was observed in primary HESC by a plaque forming assay (PFA) (Supplementary Fig. S2a) 3 days post-infection, with intracellular co-localization of the E protein and dsRNA with calreticulin and vimentin, respectively (Supplementary Fig. S2b,c). Although side-by side comparison revealed that the MR766 strain was the most efficient in terms of virus replication, only the infectious titer of Thailand 2013 was significantly lower than that of the MR766 strain.

Thus, primary HESC are targets of *in vitro* infection and actively support the replication of the hystorical MR766 strain and, more importantly, of recently obtained (2013–2016) primary ZIKV strains.

### *In vitro* decidualization of T-HESC cells upregulates ZIKV productive infection

**Figure 2:**
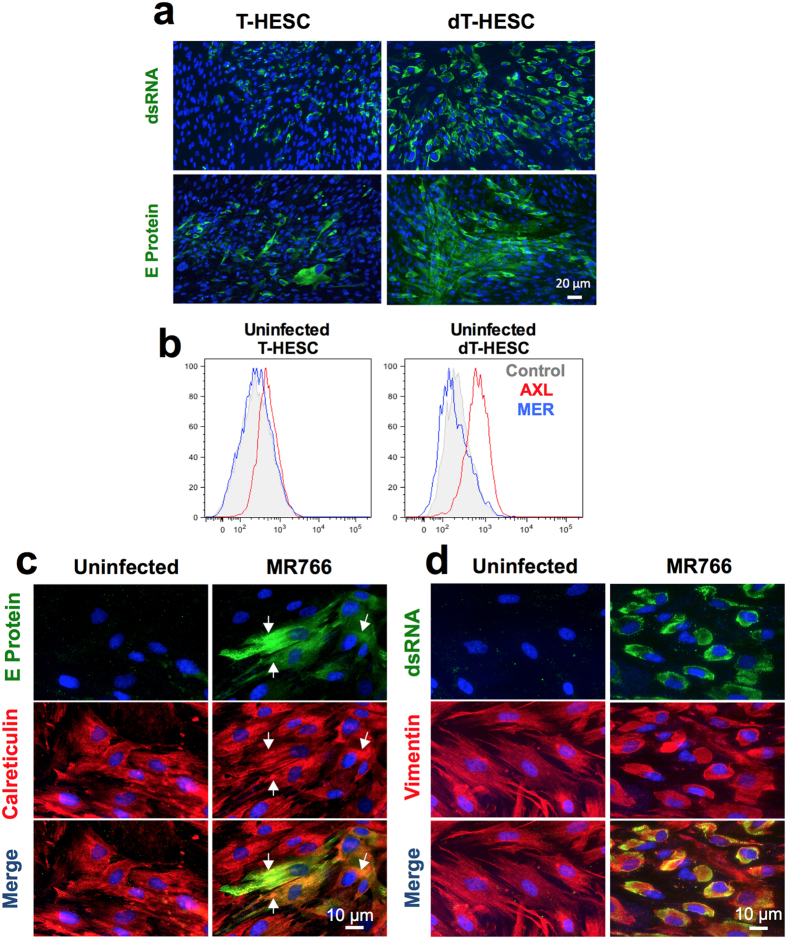
MR766 infection of unstimulated and decidualized T-HESC cell line. (**a**) T-HESC (left) and decidualized (**d**) T-HESC (right) were stained with ZIKV anti-dsRNA or E protein mAbs whereas the nuclei were stained with Hoechst. (**b**) Surface expression of AXL (red) and MER (blue) in T-HESC (left) and dT-HESC (right) was determined in uninfected cells by flow cytometry. The histograms of one experiment representative of 3 independently performed are shown. Double immunostaining for ZIKV E protein and calreticulin (**c**) or dsRNA and vimentin (**d**) in dT-HESC either uninfected or infected with MR766 at 72 h post-infection; Hoechst was used to stain nuclei. Arrows indicate localization of E protein in areas occupied by calreticulin.

In order to test whether decidualization could influence ZIKV infection, we infected the immortalized T-HESC cell line either in unstimulated conditions or following *in vitro* decidualization induced by progesterone and cAMP, as reported^27^. As shown by IFA with both anti-dsRNA and anti-E protein mAbs, the MR766 strain productively infected T-HESC. An increase of the total number of infected cells of *ca.* 2-fold was observed by IFA staining of dT-HESC *vs.* control cells (Fig. 2a).

The expression of two putative entry co-receptors for ZIKV entry, AXL and MER, was evaluated by cytofluorimetric analysis in both uninfected T-HESC and dT-HESC. While MER expression was not detectable, AXL was expressed by unstimulated T-HESC and, of note, it was upregulated in dT-HESC (RFI: 2.76 *vs.* 1.64, respectively; Fig. 2b), consistently with the higher infection efficiency observed in these experimental conditions.

As observed with primary cells, calreticulin staining in uninfected dT-HESC yielded a diffuse reticular pattern. In infected cells, it colocalized with the viral E protein (Fig. 2c). Only minor differences, such as a concentration of calreticulin in areas occupied by E protein (see arrows) were evident. Instead, staining with anti-dsRNA antibody identified punctate structures that colocalized with rearranged vimentin filaments in the perinuclear region of infected cells (Fig. 2d).

### ZIKV productive infection of T-HESC is cytopathic

**Figure 3:**
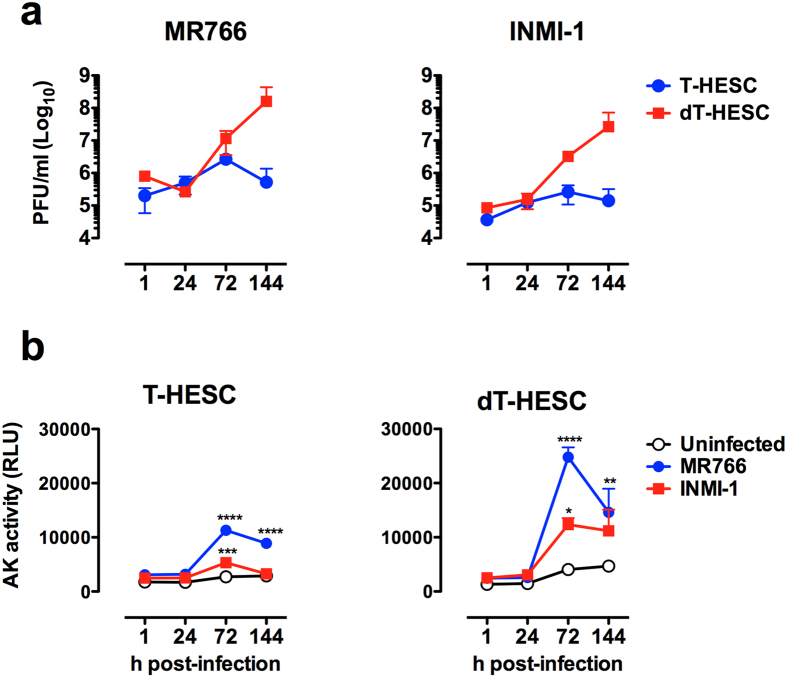
ZIKV replication and cytopathicity. (**a**) Kinetics of MR766 replication (left) and INMI-1 (right) in T-HESC (blue) and dT-HESC (red) were measured by retrotitration of culture supernatants on Vero cells by PFA. Mean ± SEM of 3 independent experiments is reported. (**b**) Kinetics of cell death were measured by the activity of cell-associated adenylate kinase (AK) released in cell culture supernatants from T-HESC (left) and dT-HESC (right). Two-way ANOVA with Bonferroni post-tests was used. *Represents statistical comparison between the viral strains and uninfected cultures (****p < 0.0001; ***p < 0.001; **p < 0.01; *p < 0.05).

We next determined the kinetics of virus replication in T-HESC and dT-HESC cells by transferring their culture supernatants on Vero cells followed by PFA. Virus replication in T-HESC of both MR766 and INMI-1 strains increased by approximately 10- and 5-fold over the levels of input virus, respectively, peaking around 96 h post-infection (Fig. 3a). Decidualization of T-HESC increased the efficiency of virus replication by up to two orders of magnitude 144 h post-infection (Fig. 3a). ZIKV infection of both untreated T-HESC and dT-HESC induced cytopathicity and cell death as determined by the levels of adenylate kinase (AK) released in the culture supernatants^28^ with kinetics similar to those of virus replication (Fig. 3b).

### ZIKV induces the expression of IFN-β and ISGs

**Figure 4:**
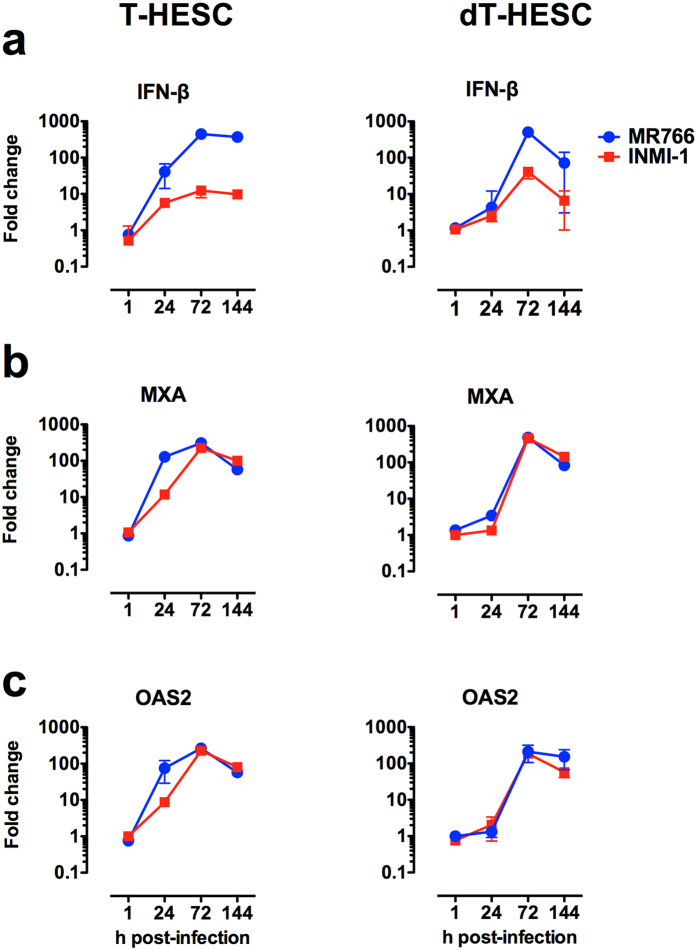
ZIKV induction of IFN-β and ISG expression in T-HESC and dT-HESC. Time course of IFN-β mRNA (**a**), MXA (**b**) and OAS2 (**c**) expression in T-HESC (left) and dT-HESC (right) quantified by RT-qPCR. The results are expressed as the fold induction of transcripts in ZIKV-infected cells relative to those of uninfected cells. The results of one experiment representative of 3 independently conducted are shown.

Since it has been reported that ZIKV infection leads to the transcription and release of IFN-β in human skin fibroblasts^29^, we evaluated the kinetics of IFN-β mRNA expression by RT-qPCR after infection of T-HESC. Indeed, higher levels of IFN-β gene transcription were detected 24 h after infection *vs.* those of uninfected cells and increased of *ca.* 300-fold 6 days after infection of T-HESC with the MR766 strain, whereas lower levels were induced upon infection with the INMI-1 strain (Fig. 4a). The virus-induced levels of IFN-β mRNA in dT-HESC were similar to those of T-HESC; however, a more rapid insurgence of IFN-β expression was observed in T-HESC *vs.* dT-HESC (Fig. 4a).

We next determined whether biologically active IFN-β was indeed secreted following ZIKV infection of T-HESC and dT-HESC cells. For this purpose, cell culture supernatants were tested on HEK-Blue IFN-α/β cells containing the secreted alkaline phosphatase (SEAP) reporter gene under the control of IFN-α/β inducible ISG54 promoter^30^. The induction of SEAP activity confirmed the presence of bioactive type 1 IFN in the supernatants of infected cells with moderately delayed kinetics in comparison to the upregulation of IFN-β mRNA observed in infected cells (Supplementary Fig. S3).

Among ISGs, myxovirus resistance protein 1 (MXA) and 2′-5′-Oligoadenylate Synthetase 2(OAS2) were previously shown to be most upregulated genes following ZIKV infection of fibroblasts^29^; therefore, we determined their kinetics of expression by RT-PCR. Both MXA and OAS2 mRNAs were indeed upregulated in ZIKV infected *vs.* uninfected cells (Fig. 4b,c, respectively) with kinetics almost superimposable to those of IFN-β mRNA expression. Of note, a delay of MXA and OAS2 expression in infected dT-HESC was observed in comparison to control untreated cells, although no quantitative differences were observed for these ISGs in cells infected with the MR766 and INMI-1 strains, in spite of their different levels of replication.

## Discussion

In the present study, we report that primary HESC are highly permissive to ZIKV productive infection by both an historic and different contemporary strains. We also showed that the T-HESC cell line, which models HESC in the proliferative phase, was permissive to ZIKV infection and supported virus replication. Infection of T-HESC strongly upregulated the expression of bioactive IFN-β and of related ISGs (MXA, OAS2). Interestingly, *in vitro* decidualization of T-HESC, increased the efficiency of ZIKV replication by up to 100-fold in association with the upregulation of the putative entry co-receptor AXL, but not of MER, suggesting a potential correlate of the increased levels of virus replication observed upon decidualization of the cell line.

Flaviviruses bind to a variety of surface molecules that serve as entry mediators or cofactors including the TAM family of tyrosine kinase receptors^31^. Among TAMs, AXL was reported to mediate ZIKV entry in dermal fibroblasts and epidermal keratinocytes^29^. Furthermore, AXL is highly expressed by neural stem cells, a privileged target of ZIKV infection in the fetal central nervous system^32^, whereas it might play a minor role as compared with other TAM entry factors in placental cells^33^. Of importance is the observation that AXL was confirmed as ZIKV entry factor in a CRISPR/Cas9 screen of HeLa cells^34^. Our results support the hypothesis of a prominent role of AXL in infection of HESC and a potential deleterious role of progesterone in favoring virus transmission to the trophoblasts, in case of pregnancy, or during sexual intercourse with an infected partner. Besides augmenting flavivirus entry^35^, AXL renders target cells more permissive to post-entry replication steps and productive infection^36^. Indeed, it has been shown in glial cells that ZIKV-triggered TAM receptor activation is associated with a marked inhibition of IFN-β expression^37^. However, in our experimental system, the kinetics of ZIKV replication well correlated with the production of active IFN-β and the consequent induction of ISGs.

As for DENV, we observed that ZIKV dsRNA associated with vimentin intermediate filaments that were rearranged in the perinuclear region of infected HESC and T-HESC^38^. This observation suggests a potential role of vimentin in ZIKV RNA amplification machinery, perhaps by stabilization of replication complexes, as described for DENV replication^38^. We also observed that ZIKV E protein was localized in the ER of infected HESC and T-HESC cells, which showed dilated *cisternae* as a sign of massive expansion of the cell secretory machinery. These observations indicate that ZIKV, similarly to DENV, assembles and accumulates its progeny virions in the ER sacs^39^ where a subset of ER-associated signal peptidase complex proteins is responsible for the proper cleavage of flavivirus structural proteins and secretion of viral particles^40^.

Very recently, ZIKV has been detected in the FRT after the onset of clinical symptoms^41^ and both male-to-female^15^,^42^ and female-to-male transmission^20^ have been documented suggesting that this vector-independent route of viral transmission might have occurred more frequently than reported. Furthermore, ZIKV presence in the FRT can foster vertical transmission from mother to fetus, as recently demonstrated in a mouse model of vaginal virus infection^13^ and previously demonstrated for other members of the *Flaviviridae* family such as hepatitis C virus, in which vertical transmission from mother to child can occur in up to 10% of pregnancies^41^. As maternal decidual cells are in direct contact with extravillous trophoblast (EVT) on the tip of anchoring villi, one possibility is that ZIKV produced by infected HESC and/or other cells present in the FRT is transmitted to EVT in early pregnancy and then enters the fetal circulation. Indeed, a recent report has provided evidence that a wide range of maternal and fetal cells isolated from first trimester placenta are highly permissive to ZIKV productive infection^12^. Furthermore, a prolonged viremic phase has been observed durign pregnancy, increasing the likelihood of mother to fetus transmission by the hematogenous route.

An unclear aspect of ZIKV pathogenesis is whether infection persists for longer in spite of its cytopathicity. If this is the case, ZIKV persistence in decidualized HESC might provide a viral reservoir for infection of cells of the *decidua basalis*, thus favoring viral transmission to chorionic villi.

Our *in vitro* findings indicate a vulnerability of the FRT to ZIKV infection, particularly upon endometrial decidualization by progesterone, as previously observed for other sexually transmitted viruses, such as herpes simplex virus and HIV^43^. As for these viruses, topical microbicides developed and formulated for vaginal use^44^ should be considered among the preventative strategies against ZIKV infection.

## Methods

### Ethics statement

Tissue samples were collected from patients undergoing gynecological surgery for benign gynecological indications at the Ospedale San Raffaele in Milan, Italy within a protocol already authorized by the Ethics Committee of the Ospedale San Raffaele. The research was conducted in accordance with all relevant legal provisions in Europe and in Italy. Research participants received appropriate information about the nature and the organization of the studies they were part of and signed an informed consent. Anonymization of personal health records, samples and data was applied, as required by the European law.

### Human Tissues

Endometrial biopsies were obtained from 7 pre-menopausal women with regular menstrual cycles (5 in proliferative phase and 2 in secretory phase) and from 1 peri-menopause woman, all undergoing histeroscopy for diagnostic purposes. The selected women did not show any evident endometrial pathology or suffer from any endocrine disorder or systemic disease and have not received any steroid treatment for at least 3 months prior to tissue collection. Uterine samples were obtained by Vacuum Aspiration Biopsy Random Assay (Vabra) (see Supplementary Information for the details of tissue culture preparation).

The immortalized human endometrial stromal cell line T-HESC, originated from normal human endometrial stromal cell immortalized with hTERT^27^, was obtained from ATCC (CRL-4003^™^). Cells were maintained in complete growth medium according to the manufacturer’s instructions. To induce T-HESC cell decidualization, stromal cells were stimulated with cAMP (0.5 mM; Sigma) and medroxyprogesterone acetate (MPA) (1 μM; Sigma) for 7 days.

### ZIKV infection

The viral strain MR766 was obtained from the European virus archive (EVAg) and expanded in Vero cells. The Brazilian 2016/INMI-1 isolate (GenBank Accession # KU991811) was obtained from an Italian individual who traveled to Brazil in January 2016. Both viral strains were expanded on Vero cells and titered in a PFA.

HESC or T-HESC cells were seeded in 24 well plastic plates at 2.5 × 10^5^/ml; cell culture medium was removed from confluent cells and was replaced with virus containing supernatant at the MOI of 10. After 4 h, the supernatant was removed and fresh culture medium (0.5 ml) was added. The kinetics of virus replication were measured in supernatants collected 1, 24, 72 and 144 h post-infection and kept frozen at −80 °C until further use (see Supporting Information).

### Indirect immunofluorescence assay (IFA) of ZIKV E protein and dsRNA

Cells grown on the cover-slip were fixed for 20 min in 4% paraformaldehyde solution (Sigma) in phosphate-buffered saline (PBS, Euroclone). Cells were permeabilized for 30 min in blocking solution, containing 0.2% Triton X-100 (Sigma) and 10% donkey serum (Sigma) and incubated overnight at 4 °C with the primary mAb in blocking solution. The following mAbs specific for Flavivirus E protein (1:200, Millipore, MAB10216), double-stranded RNA (1:300, English and Scientific Consulting Kft, Hungary), vimentin (1:300, Bioss, BS-0756R) and calreticulin (1:300, Sigma, C4606) were used. Cells were then washed with PBS and incubated for 1 h with Hoechst and either anti-mouse Alexa Fluor-488 or anti-rabbit Alexa Fluor-594 secondary Abs (1:1,000 in blocking solution, ThermoFisher Scientific). High resolution wide field fluorescence images were acquired on a Nikon Eclipse Ni-U microscope equipped with a Nikon 60x plan apo 1.40 oil or a 20x plan apo 0.75 objective and a Nikon DS-Qi2 camera.

### Plaque Forming Assay (PFA)

Vero cells were seeded in 6-well culture plates. Ten fold dilutions of virus samples were prepared in culture medium supplemented with 1% heat-inactivated FBS and 1 ml of each dilution was added to the cells. The plates were incubated for 4 h at 37 °C. Unadsorbed virus was removed and 2 ml of culture medium supplemented with 1% methylcellulose (Sigma) were added to each well, followed by an incubation at 37 °C for 6 days. The methylcellulose overlay was removed and the cells were stained with 1% crystal violet in 70% methanol. Plaques were counted and expressed as plaque-forming units per mL (PFU/mL).

### Flow Cytometry

Mouse anti-human AXL mAb (clone # 108724, MAB154) and mouse anti-human MER mAb (clone # 125518, MAB8912) were purchased from R&D Systems. Goat anti-mouse IgG secondary antibody RPE conjugated was purchased from ThermoFisher Scientific (P-852). 2 × 10^4^ T-HESC were used per condition. Samples were acquired on FACSCanto (BD) flow cytometer. Dead cells were excluded on the basis of propidium iodide and DAPI staining. All data were analyzed using FlowJo software (Tree Star). Relative fluorescent intensity (RFI) of AXL and MER expression on T-HESC was calculated dividing the mean AXL and MER fluorescence intensity by the mean fluorescence intensity of the corresponding isotype control mAb.

### Cell death detection assay

10 μl samples of culture supernatant were transferred on a half black 96 well plate (Costar). To each well, 50 μl of the adenylate kinase detection reagent (ToxiLight^®^ BioAssay, Lonza) was added and the plate was incubated for 10 min at room temperature. Luminescence was measured in a Mithras LB940 Microplate Reader (Berthold Technologies). The results were expressed as relative luminescent unit (RLU).

### RT-qPCR for IFN-β and ISGs

Total RNA was extracted from either non-decidualized or decidualized T-HESC cells by using a TRIzol Plus RNA purification kit, followed by DNase I treatment (Invitrogen). cDNA was synthesized from total RNA (1 μg) using a SuperScript first-strand synthesis system (Invitrogen) with random hexamers. SYBR green (Applied Biosystems) qPCR was performed with 50 ng of cDNA in a total volume of 25 μl with the primer pair (250 nM) as described in the Supplementary Table 1. All reactions were performed with an ABI 7700 Prism instrument (Applied Biosystems). mRNA expression was calculated by using the relative quantification method to uninfected samples normalized to human glyceraldehyde-3-phosphate dehydrogenase (GAPDH) mRNA expression.

### Statistical Analysis

Prism GraphPad software v. 4.0 (www.graphpad.com) was used for all statistical analyses. Comparison between two groups was performed using the Student’s t-test.

## Additional Information

**How to cite this article:** Pagani, I. *et al*. Human Endometrial Stromal Cells Are Highly Permissive To Productive Infection by Zika Virus. *Sci. Rep.* **7**, 44286; doi: 10.1038/srep44286 (2017).

**Publisher's note:** Springer Nature remains neutral with regard to jurisdictional claims in published maps and institutional affiliations.

## Supplementary Material

Supplementary Information
