# Supplementary material for: Human Endometrial Stromal Cells Are Highly Permissive To Productive Infection by Zika Virus
Source: Sci Rep. 2017 Mar 10;7:44286. doi: 10.1038/srep44286 (PMC5345097; doi:10.1038/srep44286)
Supplement: Supplementary Information [file srep44286-s1.doc]

## Human Endometrial Stromal Cells Are Highly Permissive To Productive Infection by Zika Virus

Isabel Pagani^1,9^, Silvia Ghezzi^1,9^, Adele Ulisse^2^, Alicia Rubio^3^, Filippo Turrini^1^,

Elisabetta Garavaglia^4^, Massimo Candiani^4,5^, Concetta Castilletti^6^, Giuseppe Ippolito^6^, Guido Poli^5,7^,Vania Broccoli^8^, Paola Panina-Bordignon^2,10*^ and Elisa Vicenzi^1,10*^

^1^Viral Pathogens and Biosafety Unit, Division of Immunology, Transplantation and Infectious Diseases, San Raffaele Scientific Institute, Milan, Italy;

^2^Reproductive Sciences Laboratory, Division of Genetics and Cell Biology, San Raffaele Scientific Institute, Milan, Italy;

^3^Division of Neuroscience, San Raffaele Scientific Institute, Milan, Italy;

^4^Obstetrics and Gynecology Unit, San Raffaele Scientific Institute, Milan, Italy;

^5^Vita-Salute San Raffaele University School of Medicine, Milan, Italy.

^6^National Institute for Infectious Diseases “Lazzaro Spallanzani”, Rome, Italy;

^7^AIDS Immunopathogenesis Unit, Division of Immunology, Transplantation and Infectious Diseases, San Raffaele Scientific Institute, Milan, Italy;

^8^National Research Council (CNR), Institute of Neuroscience, Milan, Italy;

^9^ co-first authors; ^10^ co-last authors


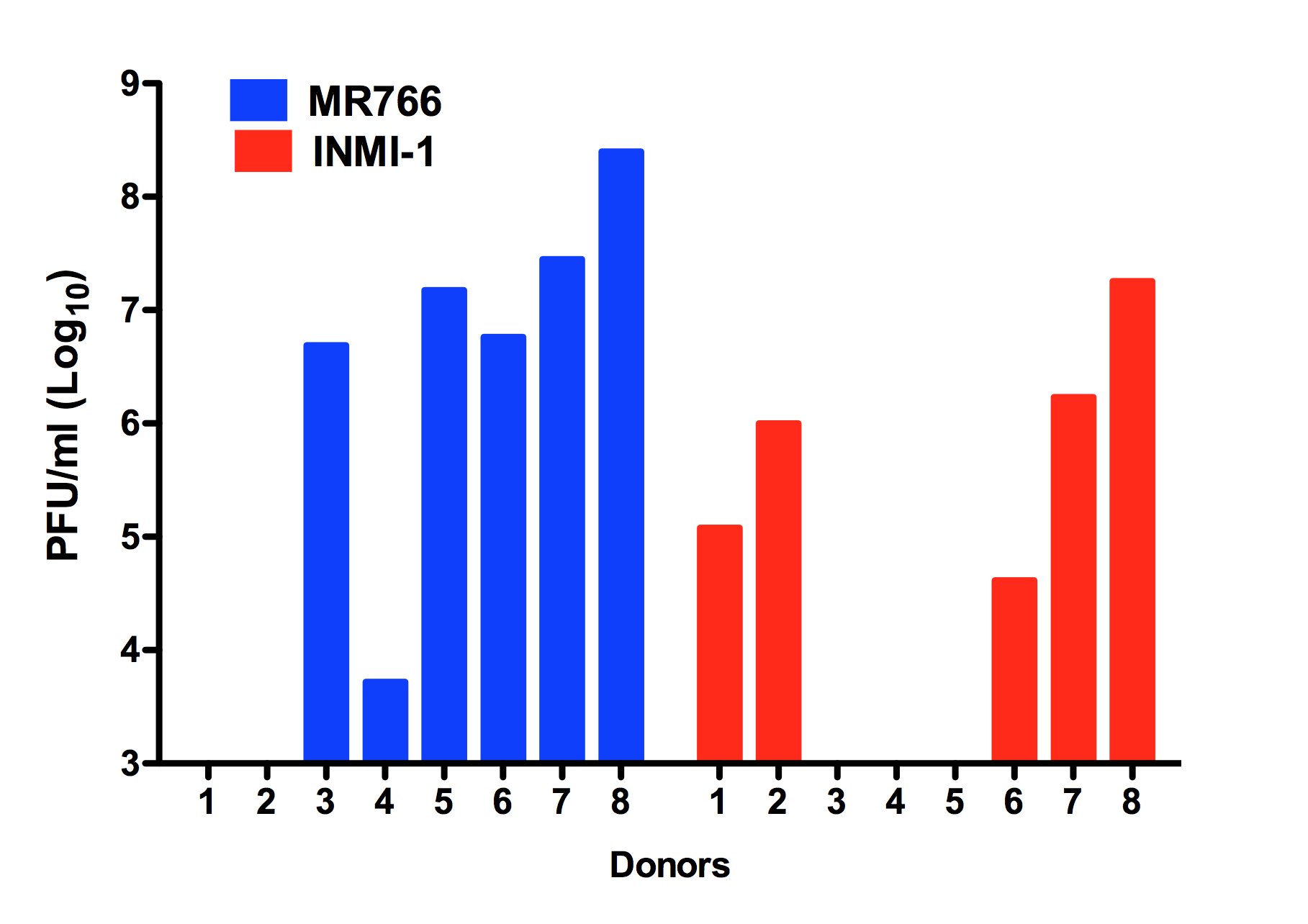
**Supplementary Fig. S1. Viral titers in HESC supernatants harvested 3 days after infection with either MR766 or INMI-1 strains**

Viral titers were determined by a PFA in VERO cells. Bars represent the plaque forming units (PFU)/ml of each donor.

**Supplementary Fig. S2. Infection of HESC with MR766, INMI-1, Puerto Rico 2015 and Thailand 2013 ZIKV strains.**

**
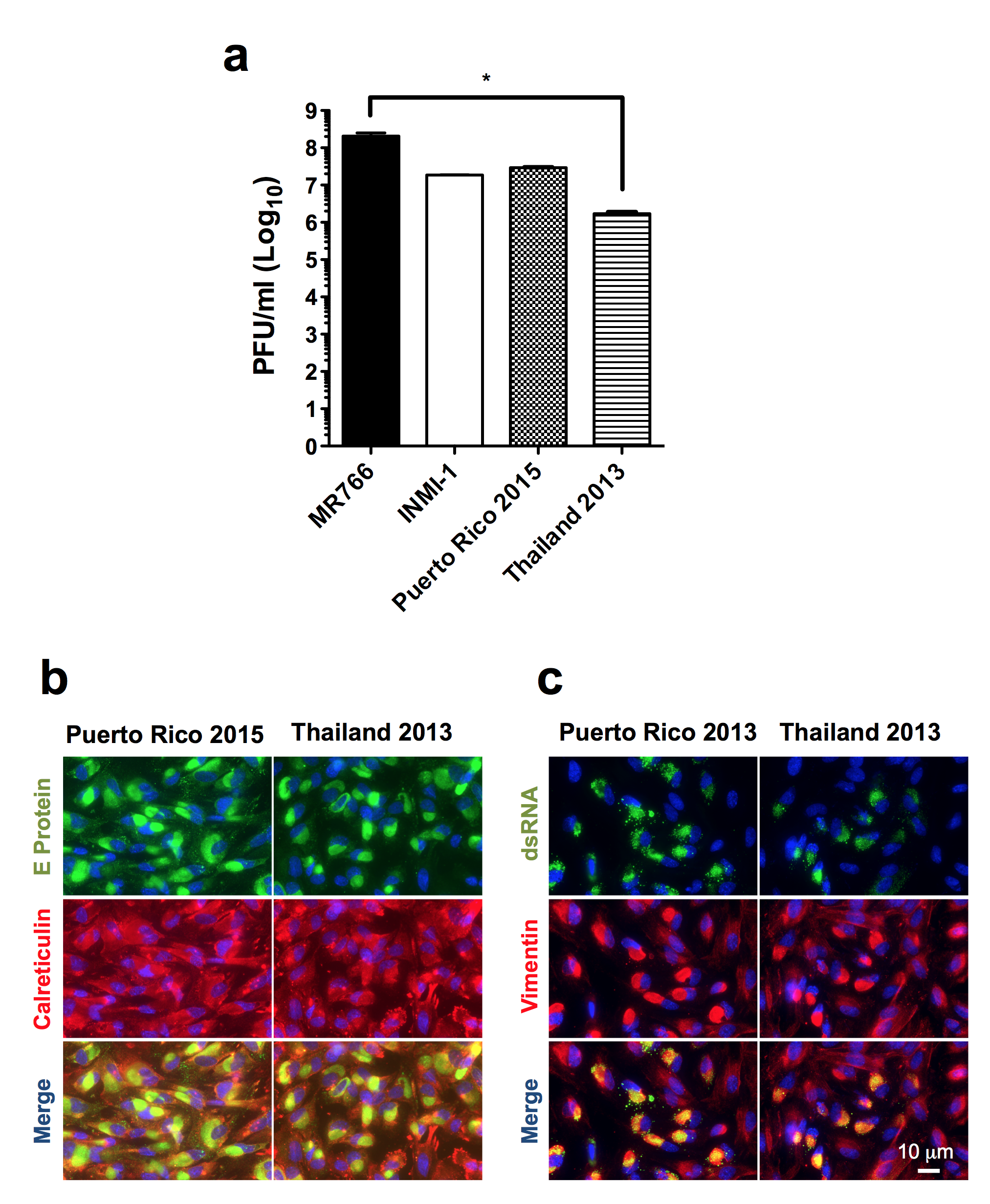
**

**(a)** Viral titers in HESC supernatant were harvested 3 days post-infection as determined by a PFA in VERO cells. The values represent the mean ± SEM of the infection of cells from one donor in duplicate cultures per condition. * represents statistical comparison between the viral strains, *, p<0.05. Double immunostaining for ZIKV-E Protein and calreticulin (**b**) or ZIKV dsRNA and vimentin (**c**) expression in HESC either mock-infected or infected with Puerto Rico 2015 and Thailand 2013 viral strains 3 days post-infection. Hoechst was used to stain nuclei.

**Supplementary Fig. S3. Time course of bioactive IFN-β release in response to ZIKV in T-HESC and dT-HESC.**

The IFN reporter HEK-BlueTM IFN-α/β cell line was used for the detection of bioactive IFN-β. Cells were incubated with 20 µl of cell supernatant for 20 h. The IFN/Alkaline Phosphatase (SEAP) activity was evaluated using the Quanti-blue substrate. The results of one experiment representative of 3 independently performed are shown.

**Supplementary Table 1. Primers used in the study**

| Target |  | 5’-3’ primer sequence |
| --- | --- | --- |
| IFN-β | For | CCAACAAGTGTCTCCTCCAAATT |
|  | Rev | GTAGGAATCCAAGCAAGTTGTAGCT |
| MxA | For | ACAACCTGTGCAGCCAGTATGA |
|  | Rev | AGCCCGCAGGGAGTCAAT |
| OAS2 | For | TGTTTGGAGGCTCTCTTGTG |
|  | Rev | CACAACTGGGTGGCATCTAC |
| GAPDH | For | CCACCCATGGCAAATTCC |
|  | Rev | TGGGATTTCCATTGATGACAAG |

**SUPPORTING EXPERIMENTAL PROCEDURES**

### *HESC cultures*. Biopsies were processed within 1 h after collection and primary cell cultures were initiated immediately after tissue processing. Endometrial tissues were dissected and gently minced under sterile conditions into small pieces (1–2 mm^3^), after washing in fresh medium to remove mucus or debris. Thereafter, they were incubated for 1 h at 37° C in a shaking water bath in RPMI 1640 (10% FBS, 1% L-Glutammine 200 mM, 2% Penicillin-Streptomycin 10.000 U/mL, Lonza) containing 0.05% collagenase (20 mg/mL, Sigma Aldrich). At the end of incubation, cells were filtered through 40 μm strainer to separate single cells from fragments of endometrial epithelial sheets and glands. The cells obtained were resuspended in 10 ml of medium, and endometrial stromal cells were separated from large clumps of epithelium during a 10 min period of differential sedimentation at single gravity. The top 8 ml medium, containing predominantly stromal cells, were then slowly removed and allowed to adhere selectively to 21 cm^2^ tissue culture dishes for 25 min at 37°C, 20% O_2_, 5% CO_2_. Selective attachment to plastic dishes was used as the final step to separate endometrial epithelial and stromal cells, with adherent cells containing a purified stromal preparation. The stromal cell fraction was established in primary culture until passage 2 in DMEM supplemented with 10% charcoal-stripped fetal bovine serum (FBS).

***Viral Isolates***. The Brazil 2016/INMI-1 isolate was passaged 6 times in Vero-E6 cells prior to experimental use in order to establish a viral stock. Puerto Rico 2015 (PRVABC59) isolate was obtained from the CDC (GenBank Accession #KU501215). The virus had been passaged 3 times in Vero cells prior to our acquisition. The Thailand 2013 viral isolate (GenBank Accession #KF993678) was obtained from a returning Canadian traveler [^1^](#_ENREF_1). All viruses were passaged 2 more time in Vero cells to establish a viral stock. Cell cultures were subjected to 3 freeze and thaw cycles and their supernatants were collected by centrifugation at 1,600 rpm per 10 min to remove cellular debris. Viral stocks were titered by a limiting dilution plaque forming assay (PFA) in Vero cells.

***Bioactive IFN quantification.*** The IFN reporter HEK-BlueTM IFN-α/β cell line was obtained from InvivoGen. Cells were seeded in 96-well plates at a density of 5x10^4^ cells/ml in 180 µl and incubated with 20 µl of cell supernatant for 20 h. The IFN/SEAP activity was evaluated using the Quanti-blue substrate according to the manufacturer’s recommendations.

**Supplemental Reference**

1 Fonseca, *K. et a*l. First case of Zika virus infection in a returning Canadian traveler*. Am J Trop Med H*y**g** 91, 1035-1038, doi:10.4269/ajtmh.14-0151 (2014).
